# Acute impact of drinking coffee on the cerebral and systemic vasculature

**DOI:** 10.14814/phy2.13288

**Published:** 2017-05-19

**Authors:** Takuro Washio, Hiroyuki Sasaki, Shigehiko Ogoh

**Affiliations:** ^1^ Department of Biomedical Engineering Toyo University Kawagoe Saitama Japan

**Keywords:** Arterial stiffness, blood pressure, caffeine, cerebral blood flow, pulsatile stress

## Abstract

Previous studies have suggested that the risk of ischemic stroke increases immediately after drinking coffee. Indeed, drinking coffee, that is, caffeine, acutely increases arterial stiffness as well as blood pressure and peripheral vascular resistance. On the other hand, it has been reported that arterial stiffening is associated with elevation in the pulsatility index (PI) of cerebral blood flow (CBF), which increases the risk of brain disease. However, the effect of drinking coffee on the PI of the CBF and its interaction with arterial stiffness remain unknown. Against this background, we hypothesized that an acute increase in arterial stiffness induced by drinking coffee augments cerebral pulsatile stress. To test this hypothesis, in 10 healthy young men we examined the effects of drinking coffee on the PI of middle cerebral artery blood velocity (MCAv) and brachial‐ankle pulse wave velocity (baPWV) as indices of cerebral pulsatile stress and arterial stiffness, respectively. Mean arterial blood pressure and baPWV were higher (*P* < 0.01 and *P* = 0.02), whereas mean MCA_V_ and mean cerebrovascular conductance index were lower upon drinking coffee (*P* = 0.02 and *P* < 0.01) compared with a placebo (decaffeinated coffee). However, there was no difference in the PI of MCAv between drinking coffee and the placebo condition. These findings suggest that drinking coffee does not increase cerebral pulsatile stress acutely despite an elevation in arterial stiffness in the systemic circulation.

## Introduction

Coffee is one of the most consumed beverages worldwide, but it has been suggested that chronic coffee drinking has a negative influence on cardiovascular function because of caffeine. Caffeine is a central nervous system stimulant of the methylxanthine class (Nehlig et al. 1992). For example, caffeine acutely increases arterial stiffness as well as arterial blood pressure and peripheral vascular resistance (Mahmud and Feely 2001). Furthermore, it has been reported that chronic coffee drinking increases the risk of cardiac arrhythmia and sudden cardiac death (Prineas et al. 1980; Smits et al. 1985; Tavani et al. 2001; Selb Semerl and Selb 2004). However, other investigations suggested that chronic coffee drinking actually reduces the risk of cardiovascular disease (Ding et al. 2015; Loftfield et al. 2015). In addition, chronic coffee drinking inhibits the accumulation of visceral fat (Mure et al. 2013), and the polyphenols found in coffee improve peripheral endothelial function (Ochiai et al. 2014). Thus, the effects of chronic coffee drinking on the cardiovascular system remain controversial. Interestingly, Mostofsky et al. (2010) demonstrated that the risk of ischemic stroke increased only immediately after drinking coffee (within 1 h) because a plasma caffeine concentration reaches peak at around 0.5 h after oral coffee intake (Teekachunhatean et al. 2013) and caffeine acutely activates the central nervous system. Thus, the effect of drinking coffee on the cardiovascular system may be time dependent, although the mechanism behind the increase in the risk of ischemic stroke immediately after drinking coffee is still unknown.

It has been suggested that an elevation of either brachial‐ankle pulse wave velocity (baPWV) or the pulsatility index (PI) of the cerebral blood flow (CBF), as an index of arterial stiffness or cerebral pulsatile stress, respectively, is associated with cardiovascular disease or acute stroke due to ischemic injury (Webb et al. 2012; Kim et al. 2015, 2016; van Sloten et al. 2015). Drinking coffee acutely increases the baPWV (Mahmud and Feely 2001), indicating that it would cause acute arterial stiffening in the systemic circulation. On the other hand, there is a positive correlation between baPWV and the PI of CBF (Kwater et al. 2009; Xu et al. 2012). Therefore, it is possible that systemic arterial stiffening induced by drinking coffee may increase pulsatile stress in the cerebral circulation. However, the effect of drinking coffee on the PI of the CBF and its interaction with arterial stiffness remain unknown. Against this background, we hypothesized that drinking coffee acutely increases cerebral pulsatile stress as well as arterial stiffness. To test this hypothesis, in this study, we determined the baPWV and the PI of middle cerebral artery blood velocity (MCAv) immediately after drinking coffee.

## Methods

This study was reviewed and approved by the institutional review board of Toyo University (IRB # 2015‐R‐04). In addition, all procedures conformed to the ethical guidelines of the Declaration of Helsinki. Written informed consent was obtained from all participants prior to the study. Ten healthy men (21 ± 0.3 years, 171 ± 6 cm in height, and 60 ± 3 kg in weight; mean ± SD) volunteered for this study. All participants were free of any known cerebrovascular, cardiovascular, or pulmonary disorders and were not taking any prescribed medication. The participants were also not habitual coffee consumers (mean consumption <100 mg of caffeine per day). They were asked to abstain from caffeinated beverages and to avoid strenuous exercise for 24 h before the experiment.

### Experimental protocol

Each participant underwent two different conditions in the experimental protocol at random and blind approach: caffeinated (Coffee) and decaffeinated coffee intake (Placebo). Each condition was performed on a different day. The average of days between first and second visit was 4 ± 2 day. During each experimental condition, after a 10‐min baseline measurement, each participant drank 250 mL of caffeinated (containing approximately 150 mg of caffeine) or decaffeinated coffee (containing approximately 4.5 mg of caffeine) at the same temperature (40°C). The participants were seated in a semirecumbent position in a reclining seat and rested quietly for 60 min after caffeinated or decaffeinated coffee intake. We measured hemodynamic variables throughout the experiment and baPWV at 30 and 60 min after caffeinated or decaffeinated coffee intake in a supine position because a plasma caffeine concentration reaches to peak at 0.44 h after oral coffee intake (Teekachunhatean et al. 2013). Each experimental protocol (Coffee and Placebo) was performed at least 2 h after a light meal in order to reduce the effect of individual diet on cerebral and peripheral vasculature.

### Measurements

Heart rate was measured using a lead II electrocardiogram (bedside monitor, BMS‐2401; Nihon Kohden, Japan). Systolic (SBP), diastolic (DBP), and mean arterial blood pressures (MAP) were continuously monitored using finger photoplethysmography (Finapres Medical Systems BV, Netherlands). MCAv was measured using transcranial Doppler ultrasonography (TCD, Multidop T; DWL, Sipplingen, Germany). A 2‐MHz Doppler probe was placed over the left temporal window and fixed with an adjustable headband. End‐tidal partial pressure of carbon dioxide (P_ET_CO_2_) was sampled using a leak‐free mask and measured using a gas analyzer (AE‐310S; Minato Medical Science Co., Osaka, Japan). The baPWV was measured in a supine position before and at 30 and 60 min after drinking coffee using an automated polygraph apparatus (form PWV/ABI; Omron‐Colin, Kyoto, Japan). An electrocardiogram (ECG), a phonocardiogram (PCG; using a microphone placed on the left edge of the sternum), and arterial pressure waveforms of the right brachial and posterior‐tibial arteries (using air‐plethysmographic sensors) were simultaneously recorded. Characteristic points of ECG, PCG, and pressure waveforms were automatically detected using a band‐pass filter. Participants changed their position from seated upright to supine and took 15 min of supine rest for the baPWV measurement, as previously reported (Sugawara et al. 2005; Kim et al. 2012; Shimawaki et al. 2015). After this measurement, participants changed their position from supine to seated upright and took a rest for measuring hemodynamic variables.

### Data analysis

The cerebrovascular conductance index (CVCi) was calculated by dividing mean MCAv by MAP. Gosling PI was calculated automatically as follows: (systolic MCAv − diastolic MCAv)/mean MCAv. The 1‐min average of hemodynamic variables was calculated before and at 30 and 60 min after drinking coffee. The baPWV was calculated using the following equation:

baPWV=(Lha−Lhb)/Tba

where Lha is the arterial path length from the aortic annulus to the midpoint of the right ankle cuff estimated using the subject's height [0.8129 ×  height (in cm) + 12.328], Lhb is the arterial path length from the aortic annulus to the midpoint of the right brachial cuff estimated using the subject's height [0.2195 ×  height (in cm) − 2.0734], and Tba is the ‘foot‐to‐foot’ time interval between brachial and posterior‐tibial arterial pressure waves.

### Statistical analysis

Data were analyzed using two‐way repeated measures analysis of variance (condition × time; SPSS 22; IBM, Tokyo, Japan). Following the detection of a significant interaction, post hoc Bonferroni tests were employed to identify the differences. All values are expressed as mean ± SD. The statistical significance for all two‐tailed tests was established at *P* < 0.05.

## Results

**Figure 1:**
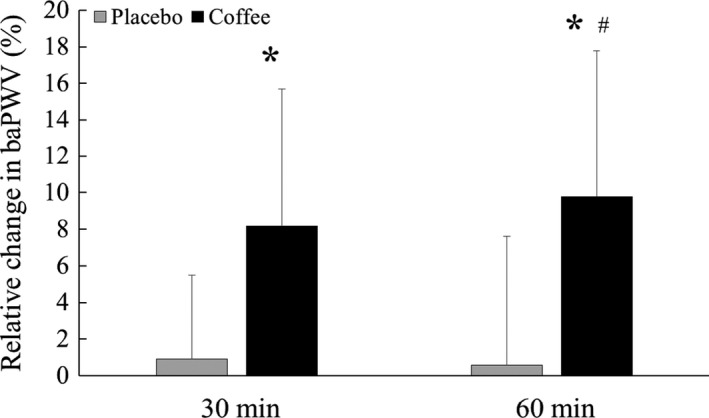
The relative change in brachial‐ankle pulse wave velocity (baPWV) from the baseline at 30 and 60 min after drinking decaffeinated (Placebo) and caffeinated coffee (Coffee). Values are mean ± SD. *Different from baseline (*P* < 0.05), #Different from Placebo (*P* < 0.05).

**Figure 2:**
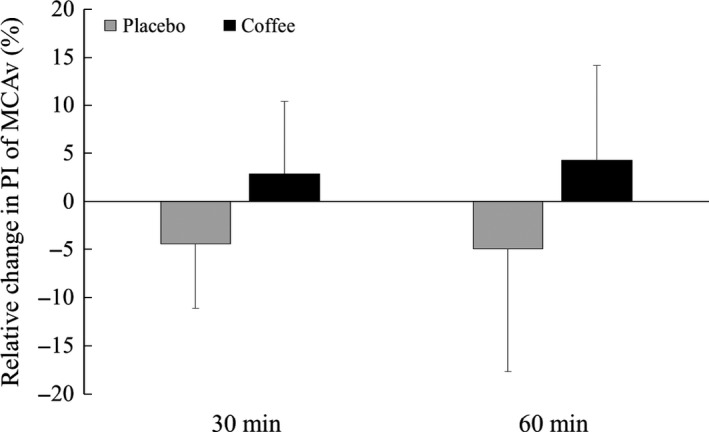
The relative change in pulsatility index (PI) of middle cerebral blood flow velocity (MCAv) from the baseline at 30 and 60 min after drinking decaffeinated (Placebo) and caffeinated coffee (Coffee). Values are mean ± SD.

At resting baseline, there was no difference in all hemodynamic parameters between Placebo and Coffee conditions. MAP and SBP were higher at 30 and 60 min after drinking in the Coffee condition than in the Placebo condition (Table 1), whereas the baPWV increased from the baseline value at 30 and 60 min after drinking coffee and change in baPWV from the baseline was larger than that of Placebo at 60 min (Fig. 1). In addition, mean MCAv, diastolic MCAv, MCA CVCi, and P_ET_CO_2_ decreased at 30 and 60 min after drinking coffee; however, the Coffee condition did not change pulse MCAv and the PI of MCAv (Table 1 and Fig. 2).

**Table 1:** Hemodynamic variables at baseline, 30 min, and 60 min after drinking decaffeinated (Placebo) and caffeinated coffee (Coffee)

	Placebo			Coffee		
	base	30 min	60 min	base	30 min	60 min
HR (bpm)	68.1 *±* 13.3	64.4 *±* 13.4	66.3 *±* 15.3	73.6 *±* 10.1	68.7 *±* 12.4	69.4 ± 14.4
SBP (mmHg)	114.3 ± 5.2	117.9 ± 7.3	119.6 ± 7.5^a^	117.2 ± 6.7	130.6 ± 8.5^a^^,^ b	134.7 ± 8.9^a^^,^ b
DBP (mmHg)	69.1 ± 6.0	72.0 ± 7.0	76.7 ± 9.5^a^	66.8 ± 4.9	78.3 ± 6.7^a^^,^ b	80.4 ± 6.1^a^
MAP (mmHg)	84.2 ± 5.1	87.2 ± 6.1	91.0 ± 7.9^a^	83.6 ± 4.8	96.7 ± 6.8^a^^,^ b	98.5 ± 5.8^a^^,^ b
Pulse pressure (mmHg)	45.2 ± 3.9	45.8 ± 6.1	42.9 ± 6.5	50.4 ± 4.8	52.3 ± 3.4	54.3 ± 7.9
Systolic MCAv (cm/s)	88.8 ± 10.9	86.7 ± 14.4	83.6 ± 12.9	88.9 ± 13.2	78.6 ± 14.0	77.2 ± 11.7
Diastolic MCAv (cm/s)	36.4 ± 5.2	36.8 ± 6.1	36.2 ± 6.1	37.0 ± 4.5	32.7 ± 5.2^a^	32.1 ± 3.7^a^
Mean MCAv (cm/s)	53.8 ± 6.2	53.5 ± 7.5	52.1 ± 7.4	54.3 ± 6.6	48.0 ± 7.6^a^	47.1 ± 5.8^a^
Pulse MCAv (cm/s)	52.4 *±* 7.9	49.9 ± 11.7	47.3 *±* 8.9	51.9 ± 10.3	45.9 ± 9.4	45.1 ± 8.7
MCA PI	0.99 ± 0.10	0.95 ± 0.15	0.94 ± 0.11	0.96 ± 0.10	0.98 ± 0.07	0.99 ± 0.07
MCA CVCi (cm/s/mmHg)	0.65 ± 0.07	0.61 ± 0.10	0.57 ± 0.10^a^	0.66 ± 0.09	0.50 ± 0.06^a^^,^ b	0.48 ± 0.06^a^^,^ b
baPWV (cm/s)	1056 ± 87	1067 *±* 121	1064 ± 134	1030 ± 82	1117 ± 143^a^	1131 ± 126^a^^,^ b
petCoz (mmHg)	38.6 ± 2.2	38.7 ± 1.8	38.3 ± 1.6	38.3 ± 2.3	37.0 ± 1.9^a^^,^ b	36.1 ± 2.7^a^^,^ b

Values are mean ± SD (*n* = 10). HR, Heart rate; SBP, systolic blood pressure; DBP, diastolic blood pressure; MAP, mean arterial pressure; MCA, middle cerebral artery; MCAv, MCA blood velocity; MCA PI, pulsatility index of MCAv; MCA CVCi, MCA cerebral vascular conductance index; baPWV, brachial‐ankle pulse wave velocity; P_ET_CO_2_, end‐tidal partial pressure of carbon dioxide.

a Different from baseline (*P* < 0.05).

b Different from Placebo (*P* < 0.05).

## Discussion

In contrast to our hypothesis, the pulse MCAv and the PI of MCAv did not increase immediately after drinking coffee, despite an increase in baPWV. These findings suggest that drinking coffee causes acute systemic arterial stiffening, but does not increase pulsatile stress in the cerebral circulation. Therefore, the acute increase in the risk of an ischemic stroke immediately after drinking coffee may not be associated with pulsatile stress for the cerebral vasculature.

Previous studies have reported that drinking coffee acutely increases the arterial blood pressure, vascular resistance, and arterial stiffness (Mahmud and Feely 2001). Similarly, in this study, MAP and baPWV increased immediately after drinking coffee (Table 1 and Fig. 1). These acute physiological responses to drinking coffee are considered to be associated with cardiovascular diseases. Indeed, it has been suggested that a transient effect of drinking coffee might represent a trigger for the first nonfatal myocardial infarction (Baylin et al. 2006). Moreover, the previous study (Mostofsky et al. 2010) has reported an increase in the risk of an ischemic stroke within 1 h after drinking coffee. Thus, the acute increase in systemic arterial pressure and arterial stiffening after drinking coffee may have a negative effect on cerebrovascular and cardiovascular function (Mahmud and Feely 2001; Tavani et al. 2001; Selb Semerl and Selb 2004). Indeed, there is a positive correlation between baPWV and the PI of CBF (Kwater et al. 2009; Safar et al. 2012; Xu et al. 2012), suggesting that systemic arterial stiffening augments the effects of pulsatile stress on the cerebral microvasculature (Lee et al. 2000).

It has been suggested that an increase in pulsatile stress causes microvascular damage (Webb et al. 2012; Tarumi et al. 2014). The presence of silent white matter lesions, a subclinical form of ischemic brain damage, is associated with high cerebrovascular pulsatility (Sierra et al. 2004). Moreover, an increase in both central and peripheral pulse pressures augments the PI of MCAv in patients with acute ischemic stroke (Kim et al. 2015). It has been reported that drinking coffee acutely increases baPWV (Mahmud and Feely 2001); therefore, we expected that the pulsatile stress of the cerebral circulation would increase after drinking coffee, which is associated with an increased risk of cerebrovascular disease. In contrast, however, drinking coffee did not increase pulsatile stress in the cerebral circulation, despite an increase in cerebrovascular resistance and systemic arterial stiffness. One possible mechanism behind this lack of alteration in the PI of MCAv involves the response of arterial pulse pressure to drinking coffee. Unexpectedly, arterial pulse pressure and pulse MCAv were unchanged after drinking coffee (Table 1).

The diet may affect vascular characteristics. For example, sodium intake was negatively correlated with arterial stiffness, while potassium intake was positively correlated with augmentation index (Garcia‐Ortiz et al. 2012). Thus, in this study, all experimental protocols were performed at least 2 h after a light meal. Thus, the protocol of this study may reduce diet influence on cerebral and peripheral vasculature. However, the result of this study may be modified by different experiment time and different diet (e.g., morning vs. evening or before lunch vs. after lunch, etc.). We need further investigations regarding this important and interesting question.

Drinking coffee also has a direct effect on the cerebral vasculature and subsequently causes acute cerebral vasoconstriction (Table 1). This cerebral vasoconstriction is caused by caffeine‐induced blockade of adenosine receptors (Ragab et al. 2004; Addicott et al. 2009; Sasaki et al. 2016) and it may be associated with an increase in the risk of cerebral disease (Mostofsky et al. 2010; Larsson et al. 2011). On the other hand, our previous study (Sasaki et al. 2016) demonstrated that dynamic cerebral autoregulation is enhanced after drinking coffee, indicating that drinking coffee acutely improves dynamic regulation of the CBF. Thus, we suggest that this improved dynamic regulation of the CBF may compensate for acute cerebral vasoconstriction induced by drinking coffee. Improved dynamic cerebral autoregulation may inhibit an increase in pulsatile stress of CBF by acutely elevating arterial stiffness. However, the physiological mechanism behind the effect of drinking coffee on the PI of MCAv remains unclear.

A methodological limitation of this study should be mentioned. First, the effect of drinking coffee on cerebral pulsatile stress was assessed from a small sample size in this study. Second, both baPWV and PI values have methodological limitations. The baPWV value may include an influence of change in arterial blood pressure. However, the previous studies (Hajjar et al. 2016) provided the validity that baPWV value can be used as an independent index with arterial blood pressure. On the other hand, the PI value of this study is not direct measurement of cerebral pulsatile stress. Also, the physiological meaning of Gosling's PI as a measure of downstream resistance is still under dispute. The PI value derived from systolic and diastolic velocity of MCA using TCD is linked to the ratio of cerebrovascular impedances (Michel and Zernikow 1998). Thus, the PI calculated by TCD data has been used widely in many previous studies as an index of pulastility. However, we cannot rule out these methodological limitations in this manuscript. Lastly, the coffee dosage administered to each participant was not proportional to the individual's body mass, indicating that the effect of coffee intake on the cerebral vasculature may not be a relative constant in this study. However, the range of body weight in all subjects was not large (55–65 kg), thus maximum influence of different relative administrated caffeine was less than 8%.

We investigated the acute effects of drinking coffee on the PI of MCAv in healthy young men. Unexpectedly, acute arterial stiffening induced by drinking coffee did not affect pulsatile stress in the cerebral circulation. These findings indicate that the acute increase in the risk of ischemic stroke observed immediately after drinking coffee may be caused by other factors rather than an elevation in pulsatile stress in the cerebral circulation.

## Conflict of Interest

No conflicts of interest for all the authors.
